# Urinary Urocortin as a Potential Non-Invasive Biomarker in Endometriosis: Exploratory Study with Histone H4

**DOI:** 10.3390/medicina61091671

**Published:** 2025-09-15

**Authors:** Bogdan Toma, Irina-Draga Caruntu, Natalia Simionescu, Mircea Onofriescu, Demetra Socolov, Ciprian Ilea, Bianca Chifu, Simona-Eliza Giusca, Andrei Daniel Timofte, Mihaela Tirnovanu, Razvan Socolov

**Affiliations:** 1 Department of Morpho-Functional Sciences I, “Grigore T. Popa” University of Medicine and Pharmacy, 700115 Iași, Romania; toma.bogdanf@gmail.com (B.T.); bianca.manole@ymail.com (B.C.); simonaelizagiusca@gmail.com (S.-E.G.); andrei-daniel.timofte@umfiasi.ro (A.D.T.); 2 “Cuza Vodă” Clinical Hospital of Obstetrics and Gynecology, 700038 Iași, Romania; mirceaonofriescu@yahoo.com (M.O.); demetrasocolov@gmail.com (D.S.); cilea1979@yahoo.com (C.I.); mihtir@yahoo.com (M.T.); 3 Romanian Medical Science Academy, 030171 București, Romania; 4 Centre of Advanced Research in Bionanoconjugates and Biopolymers, “Petru Poni” Institute of Macromolecular Chemistry, 700487 Iași, Romania; natalia.simionescu@icmpp.ro; 5 Department of Mother and Child Medicine, “Grigore T. Popa” University of Medicine and Pharmacy, 700115 Iași, Romania; socolov.razvan@gmail.com; 6 “Elena Doamna” Clinical Hospital of Obstetrics and Gynecology, 700398 Iași, Romania

**Keywords:** endometriosis, Urocortin, Histone H4, non-invasive biomarkers

## Abstract

*Background and Objectives*: Endometriosis, a complex and often underdiagnosed gynecological condition, frequently manifests with ovarian involvement, posing significant clinical challenges. Current diagnostic protocols primarily rely on invasive techniques, thus highlighting the critical need for reliable, non-invasive biomarkers. This study aimed to evaluate the diagnostic performance and clinical relevance of Urocortin and Histone H4, assessed in both serum and urine, as potential biomarkers for ovarian endometriosis. *Materials and Methods*: We implemented an exploratory study design to investigate potential biomarkers for ovarian endometriosis. The study cohort consisted of 40 women, divided into three groups: Those with histologically confirmed ovarian endometriosis are 30, those with parietal endometriosis are 5, and 5 healthy controls. Standardized ELISA protocols were employed for the quantification of Urocortin and Histone H4 in both serum and urine samples. To ensure consistency, all participants were assessed during the proliferative phase of their menstrual cycle. Finally, comparative and multivariate statistical analyses were conducted to evaluate biomarker variability in the context of relevant clinical parameters. *Results*: Serum Urocortin levels were comparable across the three groups (mean ± SD: 3.63 ± 0.41 µg/mL in ovarian endometriosis vs. 3.59 ± 0.31 µg/mL in parietal endometriosis and 3.70 ± 0.38 µg/mL in controls; *p* > 0.05). In contrast, urinary Urocortin levels were significantly elevated in patients with ovarian endometriosis (2.51 ± 1.36 µg/mL), compared to both parietal endometriosis (0.13 ± 0.04 µg/mL) and controls (0.33 ± 0.18 µg/mL; *p* = 0.001). Multivariate linear regression revealed that age, age at menarche, and disease duration accounted for 28.3% of the variance in urinary Urocortin levels (adjusted R^2^ = 0.283; *p* = 0.002). Serum Histone H4 concentrations were modestly elevated in the ovarian endometriosis group (0.49 ± 0.18 ng/mL), although no statistically significant intergroup differences were observed. Urinary Histone H4 levels showed subtle variation but lacked discriminatory value. *Conclusions*: Urinary Urocortin showed a preliminary diagnostic signal in this small exploratory cohort, whereas Histone H4 did not perform significantly. Our findings require replication in larger, multicenter, and rigorously controlled studies with validated urine normalization methods. Nonetheless, our study opens further perspectives for complementing the biomarker panel with potential non-invasive diagnostic value with new candidates.

## 1. Introduction

Endometriosis is a chronic, estrogen-dependent inflammatory condition that significantly affects women’s quality of life and fertility [1,2]. The disease frequently involves the ovaries and remains a considerable diagnostic and therapeutic challenge [1,2].

The current reliance on invasive laparoscopy for definitive diagnosis often leads to significant delays. This is a critical issue, as highlighted by global health initiatives, such as the World Endometriosis Society and the World Endometriosis Research Foundation, because delayed diagnosis contributes to disease progression and a substantial socioeconomic burden—estimated at nearly $9579 per patient annually, a cost comparable to other chronic conditions like Crohn’s disease and diabetes [1,2].

This limitation underscores the urgent need for reliable non-invasive biomarkers that could facilitate early detection and patient stratification while reducing dependence on surgery [3,4,5]. Ideally, such biomarkers should demonstrate both high sensitivity and specificity. However, progress has been hampered by the heterogeneity of disease presentation and the overlap of pathophysiological pathways [6].

Reflecting these challenges, the 2022 guidelines from the European Society of Human Reproduction and Embryology (ESHRE) explicitly discourage the clinical use of serum, endometrial, or uterine/menstrual fluid biomarkers for diagnosis in both adults and adolescents, citing insufficient robustness and reproducibility [7]. Nevertheless, research into non-invasive diagnostic tools remains highly active, with ongoing investigations into novel biomarker candidates and multi-analyte panels [8,9,10,11].

Among the emerging biomarker candidates, Urocortin—a 40-amino acid endogenous peptide of the corticotropin-releasing hormone (CRH) family—has attracted particular interest. Its three isoforms (Urocortin 1, Urocortin 2, and Urocortin 3) act as agonists of CRH-R1 and CRH-R2 receptors and share structural similarities with CRH itself [12,13,14]. Urocortin 1, in particular, is expressed in reproductive tissues such as the endometrium, ovary, and placenta, as well as in extra-neural sites including decidual stroma and placental membranes. There, it exerts autocrine and paracrine functions important for implantation, decidualization, and pregnancy maintenance [15,16,17,18,19,20,21]. Elevated Urocortin expression has been observed in ovarian endometriomas and ectopic endometrial tissue, detected through ELISA, immunohistochemistry, and qRT-PCR. These findings support its potential role as a non-invasive biomarker of endometriosis severity, though methodological variability and the absence of validated cut-off values still limit its clinical application [22,23,24,25].

Another promising candidate is Histone H4, recently identified as a potential urinary biomarker. Elevated urinary levels of Histone H4 have been reported in women with endometriosis, likely reflecting local inflammation and tissue remodeling—both hallmarks of the disease. While early studies have reported encouraging diagnostic accuracy, further validation is necessary before it can be incorporated into clinical practice [26].

For any new biomarker to prove its clinical utility, its performance must be correlated with established measures of disease severity and prognosis. The most widely adopted tools for this purpose are the revised American Society for Reproductive Medicine (rASRM) classification for staging the disease and the Endometriosis Fertility Index (EFI) for predicting postoperative fertility outcomes [27,28].

Within this scientific framework, the present study was designed as exploratory research to investigate the diagnostic potential of serum and urinary Urocortin and Histone H4 as non-invasive biomarkers in ovarian endometriosis. Furthermore, we aimed to determine their correlation with the established rASRM and EFI clinical scoring systems to assess their potential real-world value.

## 2. Materials and Methods

### 2.1. Participants

An exploratory study was conducted on a cohort consisting of 35 women surgically treated and histologically confirmed for endometriosis, divided into two groups: Ovarian localization had 30 cases (group 1), and abdominal wall involvement had 5 cases (group 2). A group of 5 healthy women undergoing routine gynecological examinations, with no evidence of endometriosis or other gynecological conditions, was used as control (group 3).

Participants were recruited between December 2020 and September 2024 from two tertiary care units: “Cuza Vodă’’ and “Elena Doamna’’ Obstetrics and Gynecology Clinical Hospitals in Iasi, Romania. All participants provided written informed consent for inclusion in this research. Ethical clearance for this study was obtained from the institutional review boards of the hospitals, as well as from the Ethics Committee of “Grigore T. Popa’’ University of Medicine and Pharmacy, Iași (approval no. 130002/24.11.2020, 390/13.01.2023, and 401/15.02.2024, respectively). All procedures conformed to international research ethics standards, including those outlined in the Declaration of Helsinki.

### 2.2. Inclusion/Exclusion Criteria

The study population was selected based on strict inclusion and exclusion criteria to ensure clinical and biological consistency. Women aged 18 to 45 years, assessed during the proliferative phase of the menstrual cycle, were eligible if they presented histologically confirmed endometriosis, typical pelvic pain symptoms (including dysmenorrhea, dyspareunia, or chronic pelvic discomfort), normal endocrine function, and preserved cognitive status. Patients were required to have no recent use (within three months) of hormonal therapies, including GnRH analogues, antagonists, or oral contraceptives. Transvaginal ultrasonography suggestive of endometriotic lesions and a prior diagnosis of infertility were also considered eligibility indicators.

Participants were excluded in cases of pregnancy, lactation, premature ovarian failure, or early menopause. Additional exclusion criteria included chronic systemic or psychiatric comorbidities, neurological disorders affecting cognition, active genitourinary infections, use of illicit substances, or any coexisting benign gynecologic disorders (e.g., adenomyosis, fibroid, benign ovarian cysts other than endometriomas). Control subjects met the same age and cycle-phase requirements, exhibited no pelvic symptoms or imaging abnormalities, and had no surgical history or clinical suspicion of endometriosis.

### 2.3. Surgery, Sample Collection, and Staging

To minimize the influence of hormonal fluctuations, all surgical procedures were scheduled during the proliferative phase of the menstrual cycle.

Biological samples were collected the day before surgery in fasting conditions at 6:00 a.m., consisting of 2 mL of peripheral venous blood and 5 mL of urine. No adverse effects were reported during the collection of blood samples, which were drawn into plain vacutainers (without anticoagulant), allowed to clot at room temperature for 30 min, and subsequently centrifuged at 2500× *g* for 10 min to obtain serum. All urine samples were collected as first-morning voids in sterile containers. Urine specimens were subjected to identical centrifugation to eliminate sediment. Both serum and urine aliquots were stored at −80° C until biochemical analysis. For the urine samples, creatinine normalization was used as a method of adjustment, given its widespread use and validation in urinary biomarker studies—as the standard approach when the patients exhibit normal function parameters. It is noteworthy that all patients demonstrated normal serum creatinine levels, both during previous routine clinical and paraclinical monitoring for endometriosis and at the defined time of urine collection for biomarker assessment.

Symptom severity was assessed using the Visual Analog Scale (VAS) for dysmenorrhea, dyspareunia, and chronic pelvic pain in three different conditions: without analgesic medication, during pharmacologic treatment, and one year after surgery. The VAS scores for dysmenorrhea, dyspareunia, and chronic pelvic pain were categorized as follows: 1–3 for mild pain, 4–6 for moderate pain, and 7–10 for severe pain [29].

The staging of endometriosis was performed after surgery using internationally recognized classification systems, including rASRM and EFI, to ensure accurate stratification and individualized assessment of disease severity.

### 2.4. Biomarker Measurement

#### 2.4.1. Urocortin Quantification

Serum and urine concentrations of Urocortin were determined using a commercial enzyme—linked immunosorbent assay (ELISA) kit, RayBio^®^ Human/Mouse/Rat Urocortin EIA (RayBiotech Inc., Norcross, GA, USA). The assay was performed according to the manufacturer’s instructions. Standards were prepared using recombinant Urocortin (0.1—1000 ng/mL). All samples and controls were assayed in duplicate. Samples were diluted accordingly in order to fit within the standard curve concentration interval. The pre-coated microplate was first coated with 100 μL of anti-Urocortin antibody and incubated for 1.5 h at room temperature with gentle agitation. After washing, 100 μL of the sample was added to each well and incubated for 2.5 h. Following subsequent washing steps, 100 μL of streptavidin-HRP solution was added and incubated for 45 min. Colorimetric detection was achieved using TMB substrate, followed by a stop solution. Optical density was measured at 450 nm using a FLUOstar^®^ Omega microplate reader (BMG LABTECH, V5.11 R2, Ortenberg, Germany). Urocortin levels were calculated based on the generated curve. For clarity and ease of visualization of the results, these values were converted to and expressed in µg/mL.

#### 2.4.2. Histone H4 Quantification

Histone H4 levels were measured using a sandwich ELISA kit (MBS2701607, MyBioSource Inc., San Diego, CA, USA), designed for quantitative in vitro analysis of Histone H4 in biological fluids. A standard curve ranging from 0.156 to 10 ng/mL was prepared using recombinant Histone H4. Each sample and standard were tested in duplicate. Following standard ELISA procedures, 100 μL of sample was added per well and incubated at 37° C for 1 h. The wells were then incubated sequentially with detection reagents A and B, each followed by appropriate washing steps. After incubation with the substrate solution in the dark at 37° C, the reaction was terminated using the stop solution, and absorbance was read at 450 nm using the same microplate reader. Results were expressed in ng/mL, based on the standard curve.

#### 2.4.3. CA-125

Serum CA-125 concentrations were determined using the Abbott Architect i2000SR system (Abbott Laboratories, Waukegan, IL, USA), employing chemiluminescent microparticle immunoassay technology, in accordance with the manufacturer’s protocol.

### 2.5. Statistical Analysis

Statistical analysis was conducted using SPSS version 18.0 (SPSS Inc., Chicago, IL, USA). Descriptive statistics (mean, median, SD, min/max) and dispersion indicators were calculated. The normality of quantitative variables was assessed using the Kolmogorov-Smirnov test (at *p* > 0.05, the null hypothesis was not rejected, indicating normal distribution), and also the Skewness test. Group comparisons were performed using Student’s *t*-test, F-test (one-way ANOVA), followed by Bonferroni post-hoc correction, Kruskal—Wallis, and chi-square test, while correlations were evaluated using Pearson’s and Spearman’s coefficients. No additional corrections were applied for correlation analyses (Pearson’s and Spearman’s coefficients), due to the exploratory nature of the study based on the small sample size, and also for regression models and ROC curve evaluations, in line with standard practice. Statistical significance was set at one-sided *p* < 0.05.

## 3. Results

### 3.1. Baseline Parameters

The age distribution across all study groups was comparable, with closely aligned mean and median values suggesting a demographically homogeneous population. Group 1 had a mean age of 33.60 ± 5.14 years, followed by group 2 at 31.20 ± 3.90 years, and group 3 at 30.40 ± 4.78 years, with no statistically significant differences (Table 1). Age of menarche ranged from 11 to 15 years, with a mean of approximating 12 years across all groups.

In group 1, 63.3% of patients reported infertility persisting for over two years (Table 1). Regarding symptom onset, 93.3% of patients with ovarian lesions experienced a delay of more than two years between initial symptoms and surgical intervention. In contrast, 80% of patients in group 2 reported symptom onset within the previous year (*p* = 0.007). Symptom onset ranged from one to ten years, with an average of 5 ± 2 years in group 1, whereas patients in group 2 reported a mean range of one-half ± 0.4 years (*p* = 0.001) (Table 1).

The assessment of symptom severity for dysmenorrhea, dyspareunia, and chronic pelvic pain using VAS showed distinct scores according to three different conditions: without analgesic medication, during pharmacologic treatment, and one year after surgery (Table 1). In the absence of analgesia, severe dysmenorrhea (VAS ≥ 7) was reported by 83% of patients with ovarian endometriosis—group 1 and 100% with parietal forms—group 2. Moderate dyspareunia (VAS 4–6) was recorded in 60% and 100% of patients in group 1 and group 2, respectively, while chronic pelvic pain affected 93% of cases in group 1 and 80% in group 2, both with moderate intensity. During pharmacologic management, dysmenorrhea persisted with VAS scores between 4 to 6 in over 95% of cases in group 1, while mild-to-moderate dyspareunia and chronic pelvic pain were observed in most patients. At one-year follow-up, 77% of patients in group 1 and 40% in group 2 reported moderate dysmenorrhea, while mild dyspareunia and chronic pelvic pain (VAS < 4) were noted in group 1 in over 70% in and 96% of cases, respectively, and in all cases in group 2.

Intraoperative findings (Table 1) confirmed all preoperative suspicions and were subsequently validated by histopathological examination in both ovarian and parietal endometriosis cases. Among patients with ovarian endometriosis, the majority (83.3%) presented with primary lesions, while 16.7% had a history of previous surgical treatment for endometriosis recurrence. Left-sided endometriomas were the most frequent (53.3%), followed by bilateral (26.7%) and right-sided (20%) localizations, with corresponding cystectomies performed. Laparoscopic surgery was the primary approach in 90% of cases, with conversion to open surgery required in 10% due to extensive adhesions and prior surgical history. Adhesiolysis was performed in nearly all patients due to obliteration of the pouch of Douglas.

### 3.2. Surgical Staging and Disease Extension

The revised American Society for Reproductive Medicine (rASRM) classification was used to assess disease severity in patients with ovarian endometriosis—group 1. Scores ranged widely, from 22 to 108, reflecting substantial interindividual variability. The average rASRM score was 50.87, notably higher than the median value of 38, indicating a skewed distribution. Based on staging criteria, 53.3% of patients were classified in stage III, while 46.7% were in stage IV.

Deep infiltrating endometriosis (DIE) was more frequently encountered in advanced stages. Specifically, 92.9% of stage IV patients and 56.3% of stage III patients presented with deep lesions, showing a statistically significant difference (*p* = 0.017). Recto-cervical and rectovaginal space involvement was observed in 50% and in 12.5% of stage IV and stage III cases, respectively (*p* = 0.023). Lesions affecting the uterosacral ligaments were noted in 92.9% of stage IV cases, compared to 50% in stage III (*p* = 0.007). In contrast, cardinal ligaments were documented in only 28.6% of stage IV cases (*p* = 0.009), and rectal invasion was confirmed in 57.1% of stage IV cases, a significantly higher rate compared to stage III (*p* = 0.001).

These findings illustrated the anatomical extension of the disease and its correlation with rASRM stage, highlighting the importance of clinical staging in evaluating the presence and distribution of deep pelvic involvement.

### 3.3. Endometriosis Fertility Index (EFI) and Postoperative Reproductive Outcomes

Postoperative reproductive outcomes were analysed in relation to EFI, which was calculated for 27 patients with preserved fertility potential following surgical treatment for ovarian endometriosis. All patients had expressed a desire to conceive in the period following surgery. EFI scores ranged from 2 to 8 and were stratified into three prognostic categories: low (EFI < 5) in 37% of patients, intermediate (EFI 5–7) in 51.9%, and higher (EFI ≥ 8) in 11.1%.

Correlating EFI scores with rASRM staging revealed a mixed contribution. Among patients with EFI < 5, half were classified in stage III and the other half in stage IV. In the intermediate EFI group (EFI 5–7), 42.9% corresponded to stage IV and 57.1% to stage III. For those with EFI ≥ 8, 33.3% were staged as rASRM IV and 66.7% as stage III. These differences were not statistically significant (*p* = 0.864).

Following surgery, 15 out of 30 patients (50%) achieved pregnancy. Among those with EFI ≥ 8, all pregnancies occurred spontaneously. In contrast, 71.4% of patients with EFI < 5 required in vitro fertilization, compared to 40% in the intermediate EFI group, showing a statistically significant association between EFI score and the method of conception (*p* = 0.045). Of the pregnancies achieved, 60% resulted in term live births, 20% ended in miscarriage, and 20% were ongoing at the time of follow-up.

### 3.4. Serum Urocortin and Histone H4 Levels

**Figure 1:**
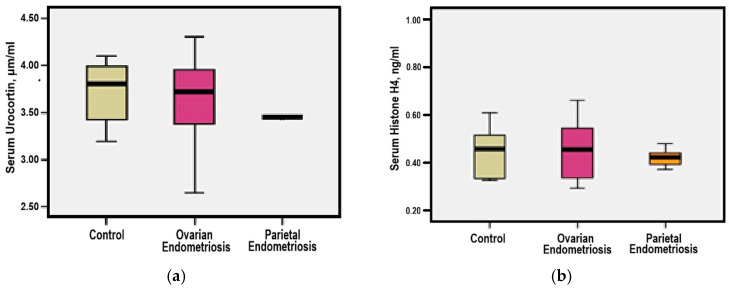
(**a**) Comparative mean serum Urocortin levels among study groups; (**b**) Comparative mean serum Histone H4 levels among study groups.

Serum Urocortin was normally distributed, as reflected by a close alignment between median (3.64 μg/mL) and mean values (3.68 μg/mL) and *p* values for the Kolmogorov-Smirnov test = 0.200, supporting the application of parametric statistical tests. In patients with ovarian endometriosis—group 1, serum Urocortin levels ranged from 2.65 to 4.30 μg/mL, with a mean value comparable to those observed in group 2 (3.59 μg/mL) and group 3 (3.70 μg/mL). No statistically significant differences were registered between groups (*p* = −0.828 and *p* = 0.722, respectively) (Figure 1a).

Serum Histone H4 concentrations demonstrated a homogenous distribution pattern, as indicated by the minimal difference between median (0.50 ng/mL) and mean (0.45 ng/mL) values and *p* values for the Kolmogorov-Smirnov test = 0.287, supporting the use of parametric tests for further analysis. In patients diagnosed with ovarian endometriosis—group 1, Histone H4 values ranged from 0.29 to 2.14 ng/mL, with a mean level comparable to that observed in the parietal endometriosis—group 2 (0.42 ng/mL) and control—group 3 (0.45 ng/mL). No statistically significant differences were found between groups (*p* = 0.528 and *p* = 0.641) (Figure 1b).

**Figure 2:**
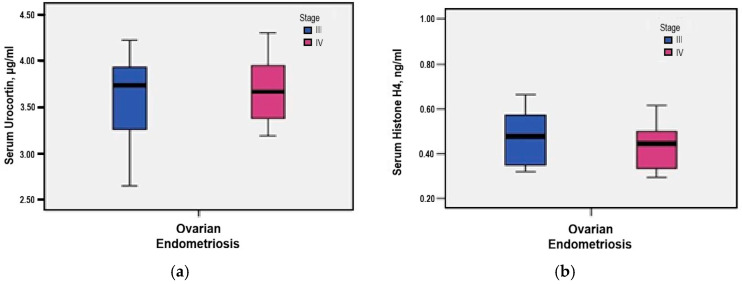
(**a**) Correlation between serum Urocortin levels and rASRM staging; (**b**) Correlation between serum Histone H4 levels and rASRM staging.

Further stratification by rASRM stage showed slightly higher serum Urocortin levels in stage IV cases compared to stage III (3.67 μg/mL *versus* 3.60 μg/mL), although this difference did not reach statistical significance (*p* = 0.621) (Figure 2a).

Further evaluation by rASRM stage showed slightly elevated serum Histone H4 levels in stage IV cases compared to stage III (0.55 ng/mL *versus* 0.51 ng/mL), although this difference did not reach statistical significance (*p* = 0.793) (Figure 2b).

### 3.5. Urinary Urocortin and Histone H4 Levels

**Figure 3:**
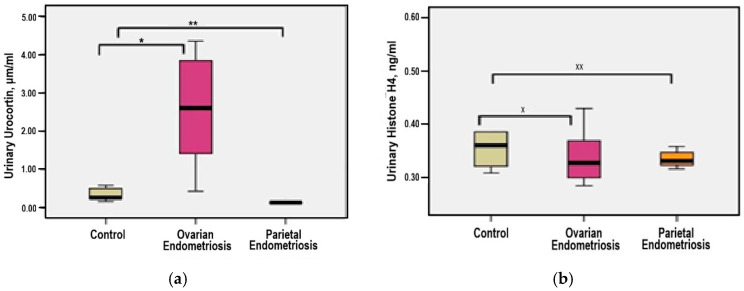
(**a**) Comparative mean urinary Urocortin levels among study groups (* *p* = 0.001 group 1 vs. group 3, ** *p* = 0.001 group 2 vs. group 3); (**b**) Comparative mean urinary Histone H4 levels among study groups (^x^ *p* = 0.115 group 1 vs. group 3, ^xx^ *p* = 0.998 group 2 vs. group 3).

The distribution of urinary Urocortin concentrations demonstrated statistical homogeneity, with a median value (1.93 μg/mL) closely aligned with the mean (2.04 μg/mL), and a skewness coefficient greater than −2, justifying the use of parametric tests for continuous variables. In patients with ovarian endometriosis—group 1, urinary Urocortin levels ranged from 0.41 to 4.23 μg/mL. Compared to the parietal endometriosis—group 2 and control—group 3, mean levels were markedly elevated (2.51 μg/mL *versus* 0.13 μg/mL and 0.33 μg/mL, respectively), with statistically significant differences observed in both comparisons (*p* = 0.001 for each) (Figure 3a).

Based on individual measurements, urinary Histone H4 levels demonstrated a homogeneous distribution, as indicated by the close alignment of median and mean values (0.34 *versus* 0.33 ng/mL) and a skewness coefficient below 2. Accordingly, statistical analysis employed significance testing for continuous variables. In the ovarian endometriosis—group 1, urinary Histone H4 concentrations ranged from 0.29 to 0.43 ng/mL. The mean levels were comparable across study groups: 0.34 ng/mL in patients with parietal endometriosis—group 2 (*p* = 0.998) and 0.39 ng/mL in controls—group 3 (*p* = 0.115), with no statistically significant differences (Figure 3b).

**Figure 4:**
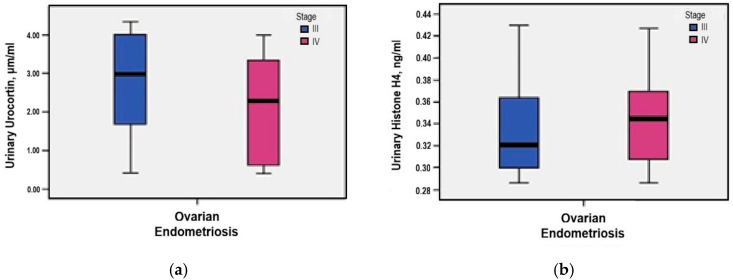
(**a**) Correlation between urinary Urocortin levels and rASRM staging; (**b**) Correlation between urinary Histone H4 levels and rASRM staging.

Further evaluation by rASRM stage showed slightly elevated urinary Urocortin levels in stage III cases compared to stage IV (2.79 μg/mL *versus* 2.19 μg/mL), although this difference did not reach statistical significance (*p* = 0.231) (Figure 4a).

Further evaluation by rASRM stage showed slightly elevated urinary Histone H4 levels in stage IV cases compared to stage III (0.35 ng/mL *versus* 0.33 ng/mL), although this difference did not reach statistical significance (*p* = 0.335) (Figure 4b).

### 3.6. Diagnostic Accuracy of Serum and Urinary Urocortin and Histone H4

**Figure 5:**
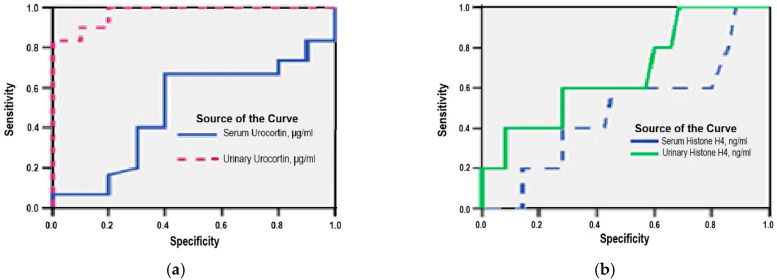
(**a**) ROC analysis of serum and urinary urocortin in ovarian endometriosis; (**b**) ROC analysis of serum and urinary histone H4 in ovarian endometriosis.

ROC curve analysis demonstrated that urinary Urocortin is a reliable non-invasive biomarker for ovarian endometriosis, with an area under the curve (AUC) of 0.973 (95% CI: 0.929–0.971; *p* = 0.001). Using a cut-off value of 2.70 μg/mL, the test achieved a sensitivity of 93% and a specificity of 80%, indicating good discriminatory power (Figure 5a).

In contrast, serum Urocortin showed limited diagnostic performance, yielding an AUC of 0.674 (95% CI: 0.458–0.890; *p* = 0.275). At a cut-off value above 4.00 μg/mL, sensitivity and specificity were 65% and 60%, respectively, suggesting a poor ability to distinguish affected from unaffected women (Figure 5a).

Urinary Histone H4 demonstrated moderate accuracy, with an AUC of 0.674 (95% CI: 0.422–0.927; *p* = 0.213). At a threshold of 0.32 ng/mL, this marker showed a sensitivity of 60% and a specificity of 43%, indicating limited clinical utility for independent diagnosis (Figure 5b).

Serum Histone H4 had the lowest predictive capacity among the tested biomarkers. ROC analysis showed an AUC of 0.538 (95% CI: 0.181–0.895; *p* = 0.811), with sensitivity and specificity both at 50% using a cut-off value of 2.70 ng/mL, reflecting a lack of diagnostic discrimination (Figure 5b).

### 3.7. Serum CA-125 Levels

**Figure 6:**
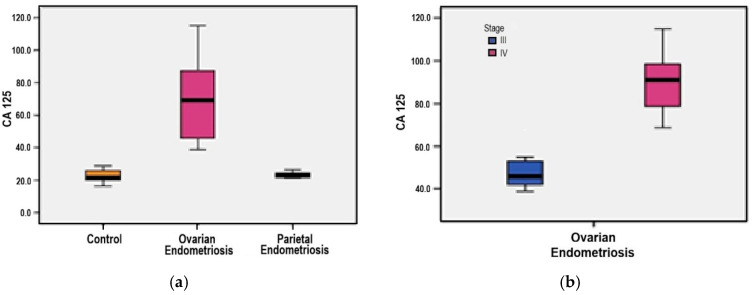
(**a**) Comparative mean Serum CA-125 levels among study groups; (**b**) Correlation between Serum CA-125 levels and rASRM staging.

The distribution of serum CA-125 levels demonstrated statistical homogeneity, with a median value (55 U/mL) closely approximating the mean (56.64 U/mL) and a skewness coefficient below 2, supporting the use of parametric tests for continuous variables. In the ovarian endometriosis group, CA-125 values ranged from 38.8 to 114.9 U/mL. Mean concentrations were remarkably higher in this group compared to both the parietal endometriosis group (67.87 U/mL versus 23.32 U/mL) and the control group (67.87 U/mL versus 22.58 U/mL), with statistically significant differences observed in both comparisons (*p* = 0.001 for each) (Figure 6a).

Further stratification by rASRM stage showed a significant elevation in serum CA-125 levels among patients with stage IV disease compared to those classified as stage III (89.44 U/mL versus 48.99 U/mL, *p* = 0.001) (Figure 6b).

### 3.8. Multivariate Regression Analysis

#### 3.8.1. Serum Urocortin

A multivariate linear regression model was applied to evaluate the potential influence of clinical parameters on serum Urocortin levels in patients with ovarian endometriosis. Among all tested variables, patient age emerged as the only independent predictor with a statistically significant contribution, explaining approximately 14.5% of the total variance in serum Urocortin concentration (adjusted R^2^ = 0.145; *p* = 0.22). The regression equation indicated a mild inverse relationship:

Urocortin=4.51−0.26×Age

When additional variables—including age at menarche, disease duration, pain intensity scores (VAS), infertility duration, and rASRM score—were included, no significant improvements in model fit were observed (*p* > 0.005 for all). The most comprehensive model, integrating all six predictors, yielded an adjusted R^2^ of 0.077 and remained statistically non-significant (*p* = 0.250), suggesting that the influence of these combined clinical parameters on serum Urocortin is limited.

#### 3.8.2. Urinary Urocortin

In contrast, multivariate analysis for urinary Urocortin revealed a more substantial association. A model including patient age, age at menarche, and disease duration explained 28.3% of the observed variance in urinary Urocortin levels (adjusted R^2^ = 0.283; *p* = 0.002), indicating a moderate predictive value for these parameters when combined.

Simpler models, such as those evaluating age alone (*p* = 0.909) or age plus age at menarche (*p* = 0.145), failed to achieve statistical significance. The addition of VAS pain score, infertility duration, or rASRM score to extended models did not enhance predictive performance and produced lower adjusted R^2^ values.

#### 3.8.3. Serum Histone H4

Multivariate regression models assessing serum Histone H4 concentrations did not identify any significant predictive relationships with the examined clinical variables. Across all tested models—including combinations of age, age at menarche, disease duration, VAS pain scores, infertility duration, and rASRM score- no statistically significant associations were detected (all *p* > 0.005).

The most inclusive model achieved only a modest adjusted R^2^ of 0.272, and the basic model including age alone resulted in negligible predictive capacity (adjusted R^2^ = 0.033; *p* = 0.806), reinforcing the lack of a reliable relationship between serum Histone H4 levels and clinical parameters in this cohort.

#### 3.8.4. Urinary Histone H4

Urinary Histone H4 levels showed a weak but borderline significant association with clinical variables. The final regression model—comprising age, age at menarche, disease duration, VAS pain scores, infertility duration, and rASRM score—explained 4.5% of the variation in the urinary Histone H4 levels (adjusted R^2^ = 0.045; *p* = 0.050).

While this result reached statistical significance, the explanatory power was limited, and individual regression coefficients were of low magnitude, indicating only a minimal clinical impact. All simpler models with fewer predictors showed non-significant results and adjusted R^2^ values below 5%, supporting the interpretation of a marginal and inconsistent association.

### 3.9. Correlation Patterns of Serum and Urinary Biomarkers in Ovarian Endometriosis

The diagnostic potential of serum and urinary Urocortin, Histone H4, and CA-125 was further evaluated through correlation analysis and clinical fertility indicators, particularly EFI.

**Figure 7:**
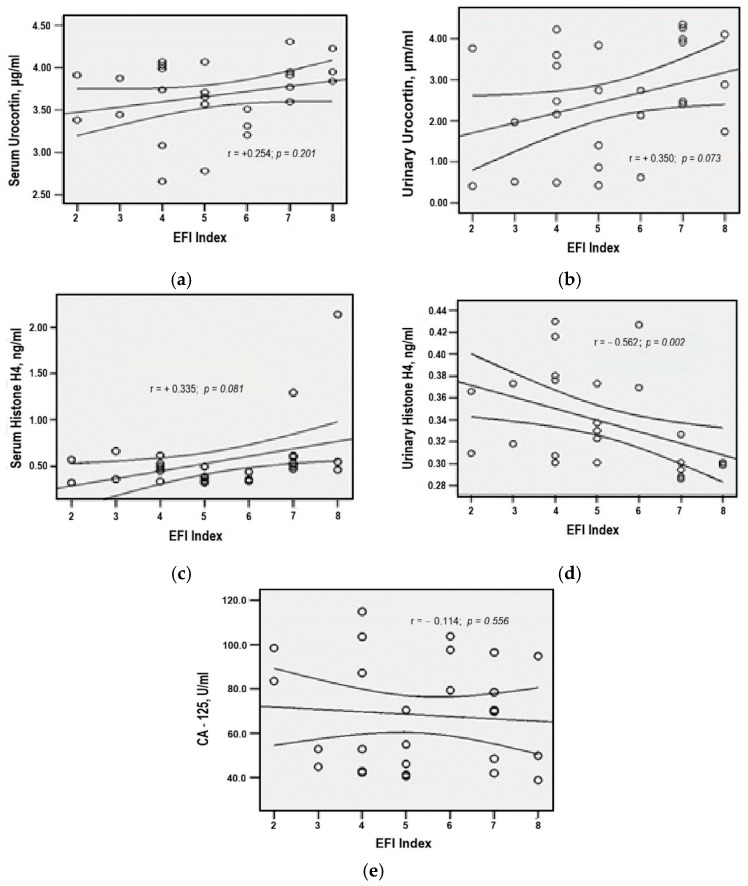
Spearman correlation between EFI Score and Urocortin (**a**,**b**), Histone H4 (**c**,**d**), and CA-125 (**e**) levels in ovarian endometriosis.

Spearman correlation analysis in the ovarian endometriosis group revealed a moderate, statistically significant inverse association between urinary Histone H4 levels and EFI scores (r = −0.562, *p* = 0.002), suggesting that lower concentrations of urinary Histone H4 were associated with more favorable reproductive outcomes (Figure 7).

Serum Histone H4 demonstrated a positive, but weak and statistically non-significant, correlation with EFI (r = +0.335, *p* = 0.081), while similar direct, low-intensity correlations were observed between EFI and both serum and urinary Urocortin (r = +0.254, *p* = 0.201)—statistical thresholds for generalizability not being met (Figure 7).

Lastly, CA-125 values exhibited a weak, non-significant inverse correlation with EFI (r = −0,114, *p* = 0.556), indicating a limited predictive relationship in this cohort (Figure 7).

In the ovarian endometriosis group, multivariate linear regression analysis revealed a statistically significant association between the rASRM score and Urocortin concentrations. Specifically, serum and urinary Urocortin levels together explained 19.7% of the rASRM score variance (adjusted R^2^ = 0.197; *p* = 0.006).

A more comprehensive model including serum and urinary concentrations of Urocortin, Histone H4, and CA-125 accounted for 69% of the variance in the rASRM score (adjusted R^2^ = 0.690; *p* < 0.001), indicating a strong multivariate association between these biomarkers and the surgical severity staging of ovarian endometriosis.

Additionally, multivariate linear regression analysis demonstrated that serum and urinary Urocortin concentrations collectively explained 16.3% of the variance in the EFI index (adjusted R^2^ = 0.163; *p* = 0.039), indicating a statistically significant association between this biomarker pair and reproductive prognosis in ovarian endometriosis.

Comparison of mean biomarker values according to EFI stratification revealed significantly elevated serum Histone H4 concentrations in patients with EFI scores ≥ 8 compared to those with EFI < 5 (1.05 *versus* 0.46 ng/mL; *p* = 0.038) and EFI scores between 5–7 (1.05 *versus* 0.50 ng/mL; *p* = 0.045) (Table 2). No other statistically significant differences were observed among the remaining biomarker comparisons across EFI subgroups (*p* > 0.05) (Table 2).

### 3.10. Predictive Biomarkers for Postoperative Conception in Ovarian Endometriosis

ROC curve analysis was performed to assess the predictive potential of serum and urinary biomarkers, as well as clinical variables, for postoperative conception in patients with ovarian endometriosis.

**Figure 8:**
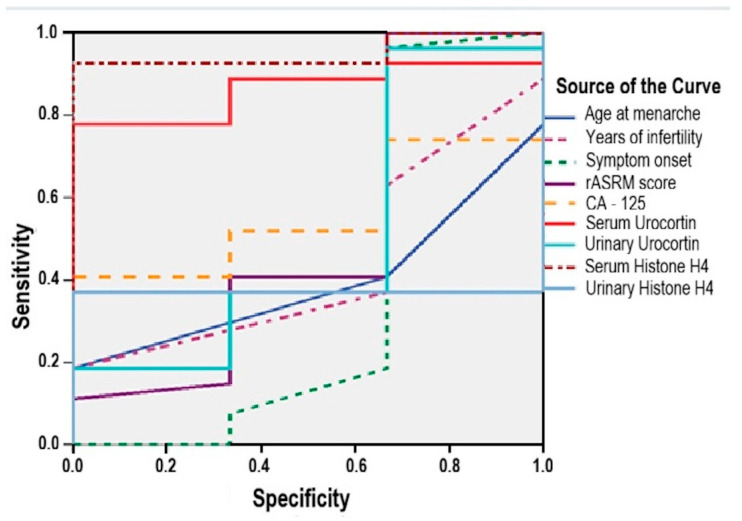
ROC Curve. Clinical predictors of postoperative pregnancy achievement.

Serum biomarkers demonstrated the following predictive value (Figure 8):

- Serum Urocortin was a significant predictor (AUC = 0.864; 95% CI: 0.728–0.999; *p* = 0.041), with a sensitivity of 85% and specificity of 67% at a cut-off value above 3.25 μg/mL;
- Serum Histone H4 showed even stronger performance (AUC = 0.951; 95% CI: 0.869–0.999; *p* = 0.012), yielding a sensitivity of 92.6% and specificity of 67% for a cut-off threshold above 0.32 ng/mL.

Conversely, several clinical and biological parameters failed to demonstrate adequate predictive capacity (AUC < 0.600), including (Figure 8):

- age at menarche (AUC = 0.395; 95% CI: 0.115–0.635; *p* = 0.557);
- infertility duration (AUC = 0.438; 95% CI: 0.136–0.740; *p* = 0.730);
- symptom duration (AUC = 0.370; 95%: 0.126–0.867; *p* = 0.468);
- rASRM score (AUC = 0.512; 95% CI: 0.088–0.936; *p* = 0.945);
- serum CA125 (AUC = 0.556; 95% CI: 0.328–0.783; *p* = 0.756);
- urinary Urocortin (AUC = 0.506; 95% CI: 0.113–0.900; *p* = 0.972);
- urinary Histone H4 (AUC = 0.370; 95% CI: 0.188–0.553; *p* = 0.468).

## 4. Discussion

The study investigated serum and urinary levels of Urocortin and Histone H4 in patients with ovarian and parietal endometriosis compared to healthy controls, using a multidimensional approach encompassing several clinico-pathological parameters. Considering the limited number of cases analyzed in our cohort, together with the limited data currently available in the literature regarding the two biomarkers under investigation at both serum and urinary levels, the present study should be regarded as an exploratory one. Within this framework, our objective was to characterize the profiles of Urocortin and Histone H4 in serum and urine, as preliminary evidence of their potential association with endometriotic lesions, without advancing statements of establishing or confirming causal relationships. Although the small sample size of the cohort (35 cases, 5 controls) limits generalizability, our findings align with current evidence regarding the diagnostic complexity of endometriosis.

Demographic characteristics confirmed a higher frequency of endometriosis among urban, educated, professionally active women in their early 30s, consistent with recent reports highlighting socioeconomic disparities in access to diagnosis and care [30,31,32].

The clinical assessment pointed out that, despite receiving hormonal therapy prior to surgery, patients still reported severe pain symptoms, particularly dysmenorrhea and dyspareunia, reflecting the persistence of symptom burden in advanced stages [33,34].

Clinical staging using rASRM classification showed a high frequency for stages III and IV of ovarian endometriosis, with deep infiltrating lesions, particularly in the uterosacral ligaments and rectovaginal space. Left ovarian localization was predominant, in line with anatomical and physiological hypotheses previously reported [35,36,37].

Postoperative follow-up showed that 50% of patients achieved pregnancy, with EFI score being an encouraging predictor of reproductive outcome. All patients with EFI ≥ 8 conceived spontaneously, while most with EFI < 5 required assisted reproduction [38,39,40]. These findings support the value of EFI for individualized fertility counselling, especially in women with advanced—stage disease [41].

We reiterate the importance of identifying new non-invasive biomarkers, as standard diagnosis for endometriosis heavily relies on invasive procedures. Thus, we focused on Urocortin and Histone H4. As we mentioned above, the current body of literature provides only a limited number of reports addressing the evaluation of these two biomarkers—whether in serum, urine, or tissue. Furthermore, the scarce evidence available in mainstream sources is predominantly derived from small cohorts, particularly when compared with studies investigating the broader clinical spectrum of endometriosis. Both these aspects sustain the exploratory nature of our research. It is worth mentioning that a PubMed database search revealed only ten original articles focusing on urocortin, with sample sizes ranging from 16 cases [42,43], 22 cases [44], 39 cases [45], 40 cases [25], 42 cases [46], 50 cases [47], 76 cases [48], 86 cases [49], to 97 cases [50]. Similarly, only four original investigations have examined histone H4 in endometriosis, with cohorts comprising 15 cases [51], 24 cases [26,52], and 31 cases [53]. Furthermore, we wish to highlight that our study investigates two biomarkers concurrently, an approach not taken by most of the studies cited below, which typically focus on a single biomarker.

Urocortin is widely expressed in the central nervous system and peripheral organs, including reproductive tissues. Urocortin plays various roles in ovarian steroidogenesis, placental maintenance, and labor initiation, and is detectable in serum, urine, and tissue biopsies, with menstrual cycle-dependent fluctuations [54,55]. In our study, serum and urinary samples for determining Urocortin levels were collected in the proliferative phase to minimize hormonal interference. Recent findings suggest its involvement in endometriosis via effects on adhesion molecules, vascular tone, and immune responses [47,55]. Increased Urocortin expression in ectopic endometrial tissue could explain the lesion implantation mechanism and inflammatory microenvironment maintenance [54,55].

Serum Urocortin is elevated in endometriosis and correlates with disease progression [48,50,55], although isolated opposing studies found no correlation with severity [49]. Our results indicated a minimal variability of serum Urocortin with comparable levels between ovarian endometriosis, parietal endometriosis, and control groups. No significant correlation was found with rASRM staging. Age was the only independent predictor of serum Urocortin levels, explaining 14.5% of the variation, suggesting a link with reproductive aging and systemic inflammation. For serum Urocortin, we found a sensitivity of 65% and a specificity of 60%. However, there is a great variability in the accuracy of Urocortin as a valuable biomarker in endometriosis, mainly due to methodological approaches, with reported sensitivity of 76.2% [46], 80% [51], 88% [25,55] and specificity of 45.7% [46], 90% [25,55].

Urinary Urocortin levels showed heterogeneity, with significantly higher values in the ovarian endometriosis group compared to the parietal endometriosis and control group (*p* = 0.001). The multivariate regression analysis suggests that clinico-demographic factors could explain 28.3% of its variance (*p* = 0.002). No differences were observed between rASRM stages III and IV. For urinary Urocortin, we found a sensitivity of 93% and a specificity of 80%, accounting for a potential diagnostic accuracy. These findings support urinary Urocortin as a candidate biomarker for ovarian endometriosis, according to the literature [55]. However, given the exploratory nature of our study, its value as a potential non-invasive biomarker in this pathology warrants validation in larger studies under a rigorous methodological design.

Histone H4, part of the nucleosome core, organizes chromatin by wrapping DNA around H2A, H2B, H3, and H4 proteins [56,57]. Under general pathological conditions, extracellular Histone H4 can bind with TLR 4 receptors, triggering cytokine release and preserving inflammation [50,58].

Post-translational modifications (acetylation, methylation) of H4 regulate gene expression, with key implications on cell cycle growth, cellular death, and angiogenesis—events involved in the pathogenic mechanism of endometriosis [59]. H4 may be released from apoptotic ectopic endometrial cells and contribute to immune dysfunction [60], its aberrant expression being linked to lesion persistence [51,59]. Despite the presence of elevated serum and urinary Histone H4 levels in women with endometriosis, their diagnostic utility is still unconfirmed [26].

Our results regarding serum Histone H4 levels indicated no significant differences between ovarian endometriosis, parietal endometriosis, and control groups (*p* > 0.05). Similarly, no association was found with rASRM staging. For serum Histone H4, we found a sensitivity of 50% and a specificity of 50%, accounting for a low diagnostic accuracy. The multivariate regression found no significant predictors, limiting its diagnostic value, consistent with existing literature [58].

Urinary Histone H4 was recently identified using proteomics, with increased levels being reported in ovarian endometriosis [26]. In our study, urinary Histone H4 levels were comparable between ovarian endometriosis, parietal endometriosis, and control groups (*p* > 0.1). Clinico-demographic factors explained only 4.5% of its variance. For urinary Histone H4, we found a sensitivity of 60% and a specificity of 43%, contrasting with existing data on a better diagnostic accuracy with reported 70% sensitivity and 80% specificity [26]. Our data support its biological relevance but question its standalone diagnostic utility.

The biological profile of the patients included in the cohort was supplemented with the serum CA-125 analysis as a routine biomarker, useful especially in advanced stages of endometriosis. Our results on serum CA-125 levels across the cohort demonstrated significantly elevated concentrations in patients with ovarian endometriosis compared to those with parietal lesions and healthy controls (*p* = 0.001). These findings support CA-125 relevance in distinguishing ovarian from extra-ovarian endometriosis or physiological conditions. A significant association was also identified between CA-125 levels and clinical staging, with markedly higher levels in stage IV than in stage III (*p* = 0.001). While helpful in advanced form, CA-125’s diagnostic performance remains limited in early-stage or mild endometriosis due to its susceptibility to cyclic hormonal changes and lesion heterogeneity [61,62]. ROC curve analysis confirmed its potential diagnostic value, with 97.1% sensitivity and 60% specificity. However, larger meta-analysis sustains a wide range of sensitivity (40–100%) and specificity (64–91%) depending on the chosen threshold [61,62,63]. In our multivariate analysis, demographic variables such as age, age at menarche, and disease duration explained 34.4% of CA-125 variance (*p* = 0.003), highlighting its relationship with hormonal maturation and chronic inflammation. When including clinical and morphological factors (VAS score, years of infertility, and rASRM stage), the model’s explanatory power increased to 63.5% (*p* = 0.001), underscoring CA-125’s integrative role in reflecting disease burden.

Translationally, integrating non-invasive biomarkers with clinical scores is essential for their validation, not only in diagnosis but also in assessing the post-surgical reproductive outcome. In our study, urinary Histone H4 showed a moderate inverse correlation with EFI (r = −0.562; *p* = 0.002), indicating a possible relevance in estimating reproductive prognosis [64,65]. Conversely, serum Histone H4, Urocortin (both serum and urinary), and CA-125 showed no statistically significant correlations with EFI, although biological trends were noted. Additionally, patients with higher EFI scores (≥8) had significantly increased serum Histone H4 levels compared to those with lower EFI, suggesting inflammatory heterogeneity depending on disease severity. However, in the context of our exploratory study, all relationships between the analyzed biomarkers and EFI must be interpreted with due caution, to avoid any overestimation of their predictive value for pregnancy outcomes—without cross-validation. Consequently, these associations require a larger sample validation before clinical application.

For rASRM classification, multivariate linear regression indicated that serum and urinary Urocortin levels explained 19.7% of the variance (Radjusted = 0.197; *p* < 0.006). When Histone H4 and CA-125 were added, the model explained 69% of the rASRM variance (Readjusted = 0.69; *p* < 0.001), highlighting the diagnostic advantage of a multipanel biomarker strategy on assessing lesion extent and, consequently, endometriosis severity.

Nonetheless, in contemporary clinical practice, the identification of predictive biomarkers or clinical parameters for postoperative fertility and recurrence in ovarian endometriosis remains a critical unmet necessity. This holds relevance for personalized therapeutic planning and long-term follow-up. The present study explored the prognostic utility of emerging serum and urinary biomarkers, along with clinical indicators, in anticipating reproductive capacity and disease recurrence.

Regarding the likelihood of achieving spontaneous pregnancy following surgical treatment, our data support significant predictive value for both serum Urocortin (*p* = 0.041) and serum Histone H4 (*p* = 0.012), with the latter showing superior sensitivity. Our findings align with recent evidence implicating these molecules in inflammatory and tissue remodelling pathways central to endometriosis pathophysiology—mechanisms that may directly influence reproductive prognosis [23,52].

Overall, our research reinforces the restrictive ability of classical assessment tools, such as established scoring systems (rASRM, EFI), based on clinical and anamnesis parameters, on one hand. On the other hand, it also underscores the limitations of standalone traditional biological biomarkers. Their diagnostic and predictive utility is often insufficient, and new biomarkers are necessary for assessing the underlying biological heterogeneity of ovarian endometriosis. Therefore, our study focusing on Urocortin and Histone H4 expression in endometriosis aligns with this current trend. We acknowledge the weaknesses of our study, such as the relatively small sample size, monocentric design, and the absence of other parameters (BMI, comorbidities), which may limit external validity.

## 5. Conclusions

Developing and validating integrative predictive models for ovarian endometriosis is crucial. These models, combining molecular biomarkers, inflammatory indices, surgical staging, and clinical history, can overcome conventional diagnostic limits for personalized management. In our exploratory study, urinary Urocortin showed a preliminary diagnostic signal in this small exploratory cohort, whereas Histone H4 did not perform significantly. Our findings require replication in larger, multicenter, and rigorously controlled studies with validated urine normalization methods. However, our study opens further perspectives for complementing the biomarker panel with potential non-invasive diagnostic value with new candidates.

## Figures and Tables

**Table 1:** Baseline parameters.

Parameter	Group 1 Ovarian Endometriosis #30	Group 2 Parietal Endometriosis #5	Group 3 Control #5
**Mean age** (years)	33.60 ± 5.14	31.20 ± 3.90	30.40 ± 4.78
**Age of menarche** (years)	≈12 (11–15)	≈12 (11–15)	≈12 (11–15)
**Infertility** > 2 years	19 (63.3%)	NA	NA
**Symptom onset** > 2 years	93.3%	NA	NA
**Mean symptom duration** (years)	5 ± 2	0.5 ± 0.4	NA
**Severity symptoms—Visual Analog Scale (#, %)**			
**No Treatment**			
Dysmenorrhea—VAS ≥ 7	25 (83%)	5 (100%)	NA
Dyspareunia—VAS 4–6	18 (60%)	5 (100%)	NA
Chronic pelvic pain—VAS 4–6	28 (93.3%)	4 (80%)	NA
**Pharmacological Treatment**			
Dysmenorrhea—VAS 4–6	29 (96.7%)	5 (100%)	NA
Dyspareunia—VAS 4–6	28 (93.3%)	2 (40%)	NA
Chronic pelvic pain—VAS 4–6	16 (53.3%)	0 (0%)	NA
**1-year follow—up**			
Dysmenorrhea—VAS 4–6	23 (76.7%)	2 (40%)	NA
Dyspareunia—VAS < 4	21 (70%)	5 (100%)	NA
Chronic pelvic pain—VAS < 4	29 (96.7%)	5 (100%)	NA
**Intra-operative aspects (#, %)**			
Endometrioma laterality: left	16 (53.3%)	NA	NA
Endometrioma laterality: right	6 (20%)	NA	NA
Endometrioma laterality: bilateral	8 (26.7%)	NA	NA
Laparoscopic approach	27 (90%)	NA	NA
Conversion to open surgery	3 (10%)	NA	NA
Adhesiolysis performed	29 (96.7%)	NA	NA
**Recurrent lesion of endometriosis (#, %)**	5 (16.7%)	NA	NA

#—number; % percentage; VAS < 4—mild pain, VAS 4–6—moderate pain, VAS ≥ 7—severe pain; NA—Not Applicable.

**Table 2:** Correlation between EFI Index and investigated biomarkers.

Biomarker	Index EFI < 55 #5	Index EFI 5–7 #14	Index EFI ≥ 8 #3
CA–125 (U/mL)	72.30 ± 28.12	67.11 ± 22.18 (*p* = 0.873)	61.13 ± 29.67 (*p* = 0.784) (*p* = 0.927)
Serum Urocortin (μg/mL)	3.62 ± 0.47	3.65 ± 0.38 (*p* = 0.079)	4.00 ± 0.20 (*p* = 0.332) (*p* = 0.371)
Urinary Urocortin (μg/mL)	2.30 ± 1.44	2.58 ± 1.37 (*p* = 0.872)	2.91 ± 1.18 (*p* = 0.782) (*p* = 0.928)
Serum Histone H4 (ng/mL)	0.46 ± 0.13	0.50 ± 0.25 (*p* = 0.961)	1.05 ± 0.95 (*p* = 0.038) (*p* = 0.045)
Urinary Histone H4 (ng/mL)	0.36 ± 0.05	0.33 ± 0.04 (*p* = 0.255)	0.30 ± 0.01 (*p* = 0.111) (*p* = 0.513)

## Data Availability

The data used to support the findings of this research are available upon request to the authors.

## References

[1] Simoens S., Dunselman G., Dirksen C., Hummelshoj L., Bokor A., Brandes I., Brodszky V., Canis M., Colombo G.L., DeLeire T. (2012). The burden of endometriosis: Costs and quality of life of women with endometriosis and treated in referral centres. Hum. Reprod..

[2] Rogers P.A., D’Hooghe T.M., Fazleabas A., Giudice L.C., Montgomery G.W., Petraglia F., Taylor R.N. (2013). Defining future directions for endometriosis research: Workshop report from the 2011 World Congress of Endometriosis in Montpellier, France. Reprod. Sci..

[3] Fassbender A., Vodolazkaia A., Saunders P., Lebovic D., Waelkens E., De Moor B., D’Hooghe T. (2013). Biomarkers of endometriosis. Fertil. Steril..

[4] D’Hooghe T. (2017). Biomarkers for Endometriosis.

[5] Krygere L., Jukna P., Jariene K., Drejeriene E. (2024). Diagnostic Potential of Cytokine Biomarkers in Endometriosis: Challenges and Insights. Biomedicines.

[6] Saunders P.T.K., Horne A.W. (2021). Endometriosis: Etiology, pathobiology, and therapeutic prospects. Cell.

[7] Becker C.M., Bokor A., Heikinheimo O., Horne A., Jansen F., Kiesel L., King K., Kvaskoff M., Nap A., Petersen K. (2022). ESHRE Endometriosis Guideline Group. ESHRE guideline: Endometriosis. Hum. Reprod..

[8] Višnić A., Barišić D., Jurešić G.Č., Klarić M., Šepić T.S. (2023). Concentration of total proteins in urine as a non-invasive biomarker of endometriosis. JBRA Assist. Reprod..

[9] Konrad L., Fruhmann Berger L.M., Maier V., Horné F., Neuheisel L.M., Laucks E.V., Riaz M.A., Oehmke F., Meinhold-Heerlein I., Zeppernick F. (2023). Predictive Model for the Non-Invasive Diagnosis of Endometriosis Based on Clinical Parameters. J. Clin. Med..

[10] Condous G., Gerges B., Thomassin-Naggara I., Becker C., Tomassetti C., Krentel H., van Herendael B.J., Malzoni M., Abrao M.S., Saridogan E. (2024). Non-invasive imaging techniques for diagnosis of pelvic deep endometriosis and endometriosis classification systems: An International Consensus Statement. Facts Views Vis. ObGyn.

[11] Ravaggi A., Bergamaschi C., Galbiati C., Zanotti L., Fabricio A.S.C., Gion M., Cappelletto E., Leon A.E., Gennarelli M., Romagnolo C. (2024). Circulating Serum Micro-RNA as Non-Invasive Diagnostic Biomarkers of Endometriosis. Biomedicines.

[12] Liew H.K., Huang L.C., Yang H.I., Peng H.F., Li K.W., Tsai A.P., Chen S.Y., Kuo J.S., Pang C.Y. (2015). Therapeutic effects of human urocortin-1, -2 and -3 in intracerebral hemorrhage of rats. Neuropeptides.

[13] Hillhouse E.W., Grammatopoulos D.K. (2006). The molecular mechanisms underlying the regulation of the biological activity of corticotropin-releasing hormone receptors: Implications for physiology and pathophysiology. Endocr. Rev..

[14] Florio P., Arcuri F., Ciarmela P., Runci Y., Romagnoli R., Cintorino M., Di Blasio A.M., Petraglia F. (2002). Identification of urocortin mRNA and peptide in the human endometrium. J. Endocrinol..

[15] Florio P., Vale W., Petraglia F. (2004). Urocortins in human reproduction. Peptides.

[16] Di Blasio A.M., Pecori Giraldi F., Viganò P., Petraglia F., Vignali M., Cavagnini F. (1997). Expression of corticotropin-releasing hormone and its R1 receptor in human endometrial stromal cells. J. Clin. Endocrinol. Metab..

[17] Makrigiannakis A., Zoumakis E., Margioris A.N., Theodoropoulos P., Stournaras C., Gravanis A. (1995). The corticotropin-releasing hormone (CRH) in normal and tumoral epithelial cells of human endometrium. J. Clin. Endocrinol. Metab..

[18] Makrigiannakis A., Zoumakis E., Margioris A.N., Stournaras C., Chrousos G.P., Gravanis A. (1996). Regulation of the promoter of the human corticotropin-releasing hormone gene in transfected human endometrial cells. Neuroendocrinology.

[19] Makrigiannakis A., Margioris A.N., Chatzaki E., Zoumakis E., Chrousos G.P., Gravanis A. (1999). The decidualizing effect of progesterone may involve direct transcriptional activation of corticotrophin-releasing hormone from human endometrial stromal cells. Mol. Hum. Reprod..

[20] Makrigiannakis A., Zoumakis E., Kalantaridou S., Coutifaris C., Margioris A.N., Coukos G., Rice K.C., Gravanis A., Chrousos G.P. (2001). Corticotropin-releasing hormone promotes blastocyst implantation and early maternal tolerance. Nat. Immunol..

[21] Minas V., Loutradis D., Makrigiannakis A. (2005). Factors controlling blastocyst implantation. Reprod. Biomed. Online.

[22] Iavazzo C., Baka S., Malamitsi-Puchner A. (2009). The role of urocortin in gynecological and obstetrical conditions. Arch. Gynecol. Obstet..

[23] Pergialiotis V., Tagkou N.M., Tsimpiktsioglou A., Klavdianou O., Neonaki A., Trompoukis P. (2019). Urocortin Expression in Endometriosis: A Systematic Review. Int. J. Fertil. Steril..

[24] Muramatsu Y., Sugino N., Suzuki T., Totsune K., Takahashi K., Tashiro A., Hongo M., Oki Y., Sasano H. (2001). Urocortin and corticotropin-releasing factor receptor expression in normal cycling human ovaries. J. Clin. Endocrinol. Metab..

[25] Florio P., Reis F.M., Torres P.B., Calonaci F., Toti P., Bocchi C., Linton E.A., Petraglia F. (2007). Plasma urocortin levels in the diagnosis of ovarian endometriosis. Obstet. Gynecol..

[26] Chen X., Liu H., Sun W., Guo Z., Lang J. (2019). Elevated urine histone 4 levels in women with ovarian endometriosis revealed by discovery and parallel reaction monitoring proteomics. J. Proteom..

[27] Wang W., Li R., Fang T., Huang L., Ouyang N., Wang L., Zhang Q., Yang D. (2013). Endometriosis fertility index score maybe more accurate for predicting the outcomes of in vitro fertilisation than r-AFS classification in women with endometriosis. Reprod. Biol. Endocrinol..

[28] Adamson G.D., Pasta D.J. (2010). Endometriosis fertility index: The new, validated endometriosis staging system. Fertil. Steril..

[29] Gift A.G. (1989). Visual analogue scales: Measurement of subjective phenomena. Nurs. Res..

[30] Andres M., Casagranda de Camargo P., Orlandi C., Silva M., Ferreira A., Azevedo R., Abrão M. (2024). 12,366 Surgical Outcomes of the National Training Program (PROADI) for Minimally Invasive Surgery for Endometriosis in Brazil. J. Minim. Invasive Gynecol..

[31] Kiesel L., Sourouni M. (2019). Diagnosis of endometriosis in the 21st century. Climacteric.

[32] GiglioAyers P., Ezike O., Foley C.E., Brown B.P. (2024). Demographic Correlates of Endometriosis Diagnosis Among United States Women Aged 15–50. J. Minim. Invasive Gynecol..

[33] Lessans N., Friedman S., Dior U. (2024). 12,469 The Relationship Between Clinical Presentation and Endometriosis Types. J. Minim. Invasive Gynecol..

[34] Marín Sánchez M., Carratalá Pérez O., Martínez Gómez A., Oliva Sánchez R., Nieto Díaz A. (2024). Evaluation of hopelessness in patients with endometriosis. Clín. Investig. Ginecol. Obstet..

[35] Vercellini P., Aimi G., De Giorgi O., Maddalena S., Carinelli S., Crosignani P.G. (1998). Is cystic ovarian endometriosis an asymmetric disease?. Br. J. Obs. Gynaecol..

[36] Vercellini P., Busacca M., Aimi G., Bianchi S., Frontino G., Crosignani P.G. (2002). Lateral distribution of recurrent ovarian endometriotic cysts. Fertil. Steril..

[37] Jenkins S., Olive D.L., Haney A.F. (1986). Endometriosis: Pathogenetic implications of the anatomic distribution. Obstet. Gynecol..

[38] Demir E., Soyman Z., Kelekci S. (2022). Outcomes between non-IVF and IVF treatment after laparoscopic conservative surgery of advanced endometriosis with Endometriosis Fertility Index score >3. Medicine.

[39] Hobo R., Nakagawa K., Usui C., Sugiyama R., Ino N., Motoyama H., Kuribayashi Y., Inoue M., Sugiyama R. (2018). The Endometriosis Fertility Index Is Useful for Predicting the Ability to Conceive Without Assisted Reproductive Technology Treatment after Laparoscopic Surgery, Regardless of Endometriosis. Gynecol. Obstet. Investig..

[40] Maheux-Lacroix S., Nesbitt-Hawes E., Deans R., Won H., Budden A., Adamson D., Abbott J.A. (2017). Endometriosis fertility index predicts live births following surgical resection of moderate and severe endometriosis. Hum. Reprod..

[41] Leone Roberti Maggiore U., Chiappa V., Ceccaroni M., Roviglione G., Savelli L., Ferrero S., Raspagliesi F., Spanò Bascio L. (2024). Epidemiology of infertility in women with endometriosis. Best Pract. Res. Clin. Obstet. Gynaecol..

[42] Vergetaki A., Jeschke U., Vrekoussis T., Taliouri E., Sabatini L., Papakonstanti E.A., Makrigiannakis A. (2013). Differential expression of CRH, UCN, CRHR1 and CRHR2 in eutopic and ectopic endometrium of women with endometriosis. PLoS ONE.

[43] Vergetaki A., Jeschke U., Vrekoussis T., Taliouri E., Sabatini L., Papakonstanti E.A., Makrigiannakis A. (2014). Galectin-1 overexpression in endometriosis and its regulation by neuropeptides (CRH, UCN) indicating its important role in reproduction and inflammation. PLoS ONE.

[44] Carrarelli P., Luddi A., Funghi L., Arcuri F., Batteux F., Dela Cruz C., Tosti C., Reis F.M., Chapron C., Petraglia F. (2016). Urocortin and corticotrophin-releasing hormone receptor type 2 mRNA are highly expressed in deep infiltrating endometriotic lesions. Reprod. Biomed. Online.

[45] Novembri R., Carrarelli P., Toti P., Rocha A.L., Borges L.E., Reis F.M., Piomboni P., Florio P., Petraglia F. (2011). Urocortin 2 and urocortin 3 in endometriosis: Evidence for a possible role in inflammatory response. Mol. Hum. Reprod..

[46] Tokmak A., Ugur M., Tonguc E., Var T., Moraloğlu O., Ozaksit G. (2011). The value of urocortin and Ca-125 in the diagnosis of endometrioma. Arch. Gynecol. Obstet..

[47] Yalcin S.E., Ocal I., Yalcin Y., Selim H.S., Caltekin M.D., Aydogmus H., Kelekci S. (2017). Evaluation of the Ki-67 Proliferation Index and Urocortin Expression in Women with Ovarian Endometriomas. Eurasian J. Med..

[48] Abramiuk M., Frankowska K., Kułak K., Tarkowski R., Mertowska P., Mertowski S., Grywalska E. (2023). Possible Correlation between Urocortin 1 (Ucn1) and Immune Parameters in Patients with Endometriosis. Int. J. Mol. Sci..

[49] Chmaj-Wierzchowska K., Kampioni M., Wilczak M., Sajdak S., Opala T. (2015). Novel markers in the diagnostics of endometriomas: Urocortin, ghrelin, and leptin or leukocytes, fibrinogen, and CA-125?. Taiwan. J. Obstet. Gynecol..

[50] Maia L.M., Rocha A.L., Del Puerto H.L., Petraglia F., Reis F.M. (2018). Plasma urocortin-1 as a preoperative marker of endometriosis in symptomatic women. Gynecol. Endocrinol..

[51] Xiaomeng X., Ming Z., Jiezhi M., Xiaoling F. (2013). Aberrant histone acetylation and methylation levels in woman with endometriosis. Arch. Gynecol. Obstet..

[52] Monteiro J.B., Colón-Díaz M., García M., Gutierrez S., Colón M., Seto E., Laboy J., Flores I. (2014). Endometriosis is characterized by a distinct pattern of histone 3 and histone 4 lysine modifications. Reprod. Sci..

[53] Gong Y., Liu M., Zhang Q., Li J., Cai H., Ran J., Ma L., Ma Y., Quan S. (2024). Lysine acetyltransferase 14 mediates TGF-β-induced fibrosis in ovarian endometrioma via co-operation with serum response factor. J. Transl. Med..

[54] Oki Y., Sasano H. (2024). Localization and physiological roles of Urocortin. Peptides.

[55] Pant A., Moar K.K., Arora T., Maurya P.K. (2023). Biomarkers of endometriosis. Clin. Chim. Acta..

[56] Kouzarides T. (2007). Chromatin modifications and their function. Cell.

[57] Luger K., Mäder A.W., Richmond R.K., Sargent D.F., Richmond T.J. (1997). Crystal structure of the nucleosome core particle at 2.8 A resolution. Nature.

[58] Xu J., Zhang X., Pelayo R., Monestier M., Ammollo C.T., Semeraro F., Taylor F.B., Esmon N.L., Lupu F., Esmon C.T. (2009). Extracellular histones are major mediators of death in sepsis. Nat. Med..

[59] Bedrick B.S., Courtright L., Zhang J., Snow M., Amendola I.L.S., Nylander E., Cayton-Vaught K., Segars J., Singh B. (2024). A Systematic Review of Epigenetics of Endometriosis. FS Rev..

[60] Huang E., Wang X., Chen L. (2024). Regulated Cell Death in Endometriosis. Biomolecules.

[61] Nisenblat V., Bossuyt P.M., Shaikh R., Farquhar C., Jordan V., Scheffers C.S., Mol B.W., Johnson N., Hull M.L. (2016). Blood biomarkers for the non-invasive diagnosis of endometriosis. Cochrane Database Syst. Rev..

[62] Dochez V., Caillon H., Vaucel E., Dimet J., Winer N., Ducarme G. (2019). Biomarkers and algorithms for diagnosis of ovarian cancer: CA125, HE4, RMI and ROMA, a review. J. Ovarian Res..

[63] Tian Z., Chang X.H., Zhao Y., Zhu H.L. (2020). Current biomarkers for the detection of endometriosis. Chin. Med. J..

[64] Kobayashi H., Imanaka S., Yoshimoto C., Matsubara S., Shigetomi H. (2024). Rethinking the pathogenesis of endometriosis: Complex interactions of genomic, epigenetic, and environmental factors. J. Obstet. Gynaecol. Res..

[65] Vissers G., Giacomozzi M., Verdurmen W., Peek R., Nap A. (2024). The role of fibrosis in endometriosis: A systematic review. Hum. Reprod. Update.

